# Supplementary material for: Diurnal variation of motor activity in adult ADHD patients analyzed with methods from graph theory
Source: PLoS One. 2020 Nov 9;15(11):e0241991. doi: 10.1371/journal.pone.0241991 (PMC7652335; doi:10.1371/journal.pone.0241991)
Supplement: S1 Table — Healthy controls and clinical controls (Not ADHD). (DOCX) [file pone.0241991.s001.docx]

**S1 Table**

**Actigraphic registrations in the morning and evening, 360 min (08 – 14 and 18 - 24). Healthy controls and clinical controls (Not ADHD).**

| **Healthy controls (n = 30) Not ADHD (n = 38)** |
| --- |
| **Morning Evening P d# Morning Evening P d LMM*** |
| **D T D x T** |
| **Mean 385 ±178 309 ±139 0.049 0.48 315 ± 194 257 ± 139 0.076 0.34 0.322 0.851 0.718** |
| **SD 105 ± 31 137 ± 41 0.003 0.88 126 ± 50 139 ± 51 0.263 0.26 0.090 0.504 0.513** |
| **RMSSD 86 ± 17 104 ± 31 0.014 0.72 106 ± 43 116 ± 51 0.393 0.21 0.145 0.821 0.526** |
| **RMSSD/SD 0.85 ± 0.12 0.76 ± 0.08 0.003 0.88 0.85 ± 0.12 0.84 ± 0.15 0.486 0.07 0.376 0.272 0.113** |
| **Edges 7.94 ± 2.80 6.27 ± 2.54 0.022 0.63 7.19 ± 3.50 5.87 ± 2.74 0.091 0.42 0.430 0.922 0.641** |
| **Components 104 ± 35 146 ± 58 0.001 0.88 132 ± 68 151 ± 63 0.204 0.29 0.102 0.595 0.258** |
| **Bridges 38.6 ± 8.5 25.1 ± 11.5 <0.001 1.34 36.6 ± 8.6 31.7 ± 12.1 0.074 0.47 0.041 0.174 0.018** |
| **Missing edges 309 ± 14 317 ± 19 0.062 0.48 318 ± 17 322 ± 15 0.379 0.25 0.107 0.634 0.384** |
| **Max edges 21.8 ± 5.1 21.8 ± 7.7 0.984 0.00 22.0 ± 7.7 20.5 ± 5.8 0.266 0.22 0.649 0.442 0.527** |
| **Zero edges 117 ± 34 142 ± 50 0.030 0.58 143 ± 62 154 ± 57 0.425 0.18 0.147 0.678 0.421** |
| **Ln cliques 7. 69 ± 0.68 7.33 ± 0.86 0.093 0.46 7.52 ± 1.03 7.23 ± 0.84 0.140 0.31 0.630 0.822 0.838** |
| **Sample entropy 0.98 ± 0.46 0.52 ± 0.28 <0.001 1.21 0.74 ± 0.44 0.62 ± 0.39 0.213 0.29 0.013 0.100 0.014** |

Registrations in the morning and evening are compared with paired samples t-tests

M : linear mixed model, diagnosis = ADHD vs. healthy controls, time = morning vs. evening, D = diagnosis, T = time, D x T = interaction diagnosis and time.

#d : effect size (Cohen)
