# Supplementary material for: Diurnal variation of motor activity in adult ADHD patients analyzed with methods from graph theory
Source: PLoS One. 2020 Nov 9;15(11):e0241991. doi: 10.1371/journal.pone.0241991 (PMC7652335; doi:10.1371/journal.pone.0241991)
Supplement: S2 Table — ADHD patients with (n = 24) and without cyclothymic temperament (n = 17). (DOCX) [file pone.0241991.s002.docx]

**S2 Table**

**Actigraphic registrations in the morning and evening, 360 min (08 – 14 and 18 - 24). ADHD patients with (n = 24) and without cyclothymic temperament (n = 17).**

| **CT Not CT** |
| --- |
| **Morning Evening P Morning Evening P** |
|  |
| **Mean 280 ± 134 280 ± 153 0.990 301 ± 177 326 ± 196 0.694** |
| **SD 133 ± 43 129 ± 34 0.759 129 ± 57 140 ± 50 0.523** |
| **RMSSD 109 ± 29 110 ± 32 0.952 110 ± 47 112 ± 39 0.909** |
| **RMSSD/SD 0.85 ± 0.17 0.86 ± 0.15 0.816 0.87 ± 0.14 0.81 ± 0.11 0.128** |
| **Edges 5.98 ± 2.65 6.11 ± 2.74 0.863 7.15 ± 3.36 6.43 ± 2.83 0.427** |
| **Components 145 ± 66 139 ± 49 0.742 122 ± 70 141 ± 62 0.379** |
| **Bridges 35.6 ± 12.3 27.2 ± 9.9 0.012 36.6 ± 10.6 26.2 ± 12.4 0.012** |
| **Missing edges 323 ± 14 323 ± 12 0.921 318 ± 19 318 ± 16 0.976** |
| **Max edges 21.3 ± 7.4 20.0 ± 5.4 0.506 20.1 ± 6.9 20.2 ± 5.9 0.955** |
| **Zero edges 153 ± 62 141 ± 41 0.433 132 ± 61 141 ± 53 0.656** |
| **Ln cliques 7.25 ± 0.84 7.22 ± 0.71 0.902 7.41 ± 0.98 7.29 ± 0.92 0.704** |
| **Sample entropy 0.67 ± 0.44 0.70 ± 0.44 0.684 0.89 ± 0.46 0.61 ± 0.37 0.121** |

Paired samples t-tests
