# Supplementary material for: Diurnal variation of motor activity in adult ADHD patients analyzed with methods from graph theory
Source: PLoS One. 2020 Nov 9;15(11):e0241991. doi: 10.1371/journal.pone.0241991 (PMC7652335; doi:10.1371/journal.pone.0241991)
Supplement: S3 Table — ADHD patients (n = 42/41*) and healthy controls (n = 30). Number of bridges for different choices of neighbours. (DOCX) [file pone.0241991.s003.docx]

**S3 Table**

**Actigraphic registrations in the morning and evening, 360 min (08 – 14 and 18 - 24). ADHD patients (n = 42/41*) and healthy controls (n = 30). Number of bridges for different choices of neighbours**.

| **Healthy controls ADHD** |
| --- |
| **Morning Evening P d Morning Evening P d** |
| **Neighbours** |
| **2 + 2 74.9** **± 18.7 63.1 ± 18.7 0.029 0.63 63.7 ± 22.6 59.9 ± 20.8 0.497 0.17** |
| **5 + 5 81.9 ± 15.9 65.1 ± 19.4 0.002 0.95 72.4 ± 25.6 70.6 ± 19.1 0.794 0.08** |
| **10 + 10 68.3 ± 14.1 57.3 ± 20.0 0.014 0.64 66.7 ± 21.9 62.4 ± 16.0 0.386 0.22** |
| **20 + 20 44.9 ± 13.3 38.2 ± 13.2 0.050 0.51 43.1 ± 12.5 42.6 ± 12.1 0.970 0.04** |
| **40 + 40 38.6 ± 8.5 25.1 ± 11.5 <0.001 1.34 36.1 ± 11.5 26.8 ± 10.9 <0.001 0.83** |
| **80 + 80 64.6 ± 10.0 37.1 ± 21.5 <0.001 1.64 52.9 ± 19.4 42.4 ± 20.5 0.019 0.53** |
