# Supplementary material for: Diurnal variation of motor activity in adult ADHD patients analyzed with methods from graph theory
Source: PLoS One. 2020 Nov 9;15(11):e0241991. doi: 10.1371/journal.pone.0241991 (PMC7652335; doi:10.1371/journal.pone.0241991)
Supplement: S4 Table — (DOCX) [file pone.0241991.s004.docx]

**S4 Table**

**Effect of gender on actigraphic registrations in the morning, 360 min (18 – 24) using analysis of covariance (ANCOVA).**

| **The whole sample (controls, ADHD, not ADHD)** |
| --- |
| **N = 112** |
| **Mean F = 1.676 P = 0.198** |
| **SD (% of mean) F = 0.023 P = 0.881** |
| **RMSSD (% of mean) F = 0.132 P = 0.717** |
| **RMSSD/SD F = 0.020 P = 0.889** |
| **Edges F = 0.185 P = 0.668** |
| **Components F = 0.277 P = 0.600** |
| **Bridges F = 0.626 P = 0.430** |
| **Missing edges F = 0.270 P = 0.604** |
| **Max number of edges F = 0.037 P = 0.848** |
| **Nodes with zero edges F = 0.310 P = 0.579** |
| **Ln cliques F = 0.066 P = 0.798** |
| **Sample entropy*** |

For sample entropy there is a significant interaction between gender and diagnosis (F = 3.285, p = 0.041), violating the assumption of homogeneity of regression slopes, so the effect of gender cannot be calculated for this measure. For the healthy controls sample entropy is higher in females than in males, 1.13 ± 0.45 vs. 0.70 ± 0.33, p = 0.014 (t-test).
