# Supplementary material for: Diurnal variation of motor activity in adult ADHD patients analyzed with methods from graph theory
Source: PLoS One. 2020 Nov 9;15(11):e0241991. doi: 10.1371/journal.pone.0241991 (PMC7652335; doi:10.1371/journal.pone.0241991)
Supplement: S5 Table — (DOCX) [file pone.0241991.s005.docx]

**S5 Table**

**Effect of gender on actigraphic registrations in the evening, 360 min (18 – 24) using analysis of covariance (ANCOVA).**

| **The whole sample (controls, ADHD, not ADHD)** |
| --- |
| **N = 110** |
| **Mean F = 0.967 P = 0.328** |
| **SD (% of mean) F = 8.508 P = 0.004** |
| **RMSSD (% of mean) F = 9.527 P = 0.003** |
| **RMSSD/SD F = 0.004 P = 0.947** |
| **Edges F = 8.897 P = 0.004** |
| **Components F = 6.552 P = 0.012** |
| **Bridges F = 3.251 P = 0.074** |
| **Missing edges F = 2.360 P = 0.127** |
| **Max number of edges F = 6.341 P = 0.013** |
| **Nodes with zero edges F = 4.507 P = 0.036** |
| **Ln cliques F = 9.892 P = 0.002** |
| **Sample entropy F = 7.651 P = 0.007** |
