# Supplementary material for: Diurnal variation of motor activity in adult ADHD patients analyzed with methods from graph theory
Source: PLoS One. 2020 Nov 9;15(11):e0241991. doi: 10.1371/journal.pone.0241991 (PMC7652335; doi:10.1371/journal.pone.0241991)
Supplement: S6 Table — Controls and the clinical group divided according to the presence or not of ADHD. For the graph analyses the number of neighbours is 40 + 40. (DOCX) [file pone.0241991.s006.docx]

**S6 Table**

**Actigraphic registrations in the evening, 360 min (18 – 24) for males. Controls and the clinical group divided according to the presence or not of ADHD. For the graph analyses the number of neighbours is 40 + 40.**

| **Controls ADHD Not ADHD ANOVA** |
| --- |
| **(n = 10) (n = 23) (N = 19)** |
| **Mean 305 ±101 330 ± 197 261 ± 160 F (49,2) = 0.871, P = 0.425** |
| **SD (% of mean) 125 ± 35 121 ± 40 127 ± 40 F (49,2) = 0.138, P = 0.871** |
| **RMSSD (% of mean) 94 ± 22 100 ± 29 102 ± 25 F (49,2) = 0.271, P = 0.764** |
| **RMSSD/SD 0.76 ± 0.08 0.85 ± 0.12 0.82 ± 0.13 F (49,2) = 1.658, P = 0.201** |
| **Edges 6.67 ± 2.26 7.31 ± 2.83 6.47 ± 2.71 F (49,2) = 0.542, P = 0.585** |
| **Components 128 ± 48 125 ± 49 140 ± 52 F (49,2) = 0.465, P = 0.631** |
| **Bridges 29.4 ± 7.2 28.0 ± 10.5 33.5 ± 10.7 F (49,2) = 1.605, P = 0.211** |
| **Missing edges 318 ± 15 317 ± 13 320 ± 14 F (49,2) = 0.164, P = 0.850** |
| **Max number of edges 22.0 ± 7.2 21.9 ± 5.0 22.4 ± 5.8 F (49,2) = 0.049, P = 0.952** |
| **Nodes with zero edges 129 ± 39 130 ± 41 144 ± 41 F (49,2) = 0.743, P = 0.481** |
| **Ln cliques 7.49 ± 0.70 7.55 ± 0.69 7.46 ± 0.69 F (49,2) = 0.089, P = 0.915** |
| **Sample entropy 0.63 ± 0.29 0.78 ± 0.48 0.69 ± 0.46 F (49,2) = 0.499, P = 0.610** |
