# Supplementary material for: Diurnal variation of motor activity in adult ADHD patients analyzed with methods from graph theory
Source: PLoS One. 2020 Nov 9;15(11):e0241991. doi: 10.1371/journal.pone.0241991 (PMC7652335; doi:10.1371/journal.pone.0241991)
Supplement: S7 Table — Controls and the clinical group divided according to the presence or not of ADHD. For the graph analyses the number of neighbours is 40 + 40. (DOCX) [file pone.0241991.s007.docx]

**S7 Table**

**Actigraphic registrations in the evening, 360 min (18 – 24) for females. Controls and the clinical group divided according to the presence or not of ADHD. For the graph analyses the number of neighbours is 40 + 40.**

| **Controls ADHD Not ADHD ANOVA** |
| --- |
| **(n = 20) (n = 18) (n = 19)** |
| **Mean 311 ±158 259 ± 127 252 ± 119 F (54,2) = 1.066, P = 0.352** |
| **SD (% of mean) 143 ± 43 150 ± 38 151 ± 59 F (54,2) = 0.158, P = 0.855** |
| **RMSSD (% of mean) 109 ± 34 124 ± 37 130 ± 65 F (54,2) = 1.031, P = 0.364** |
| **RMSSD/SD 0.76 ± 0.09 0.83 ± 0.15 0.85 ± 0.17 F (54,2) = 2.131, P = 0.129** |
| **Edges 6.06 ± 2.71 4.89 ± 1.98 5.28 ± 2.72 F (54,2) = 1.087, P = 0.344** |
| **Components 155 ± 61 159 ± 56 163 ± 72 F (54,2) = 0.082, P = 0.922** |
| **Bridges 23.0 ± 12.8 25.2 ± 11.4 30.0 ± 13.5 F (54,2) = 1.526, P = 0.227** |
| **Missing edges 317 ± 20 326 ± 14 324 ± 16 F (54,2) = 1.428, P = 0.249** |
| **Max number of edges 21.7 ± 8.1 17.9 ± 5.5 18.9 ± 5.2 F (54,2) = 1.865, P = 0.165** |
| **Nodes with zero edges 148 ± 55 154 ± 50 163 ± 70 F (54,2) = 0.321, P = 0.727** |
| **Ln cliques 7.26 ± 0.94 6.87 ± 0.77 6.99 ± 0.93 F (54,2) = 0.949, P = 0.393** |
| **Sample entropy 0.46 ± 0.26 0.51 ± 0.25 0.55 ± 0.31 F (54,2) = 0.460, P = 0.634** |
