# Supplementary material for: Diurnal variation of motor activity in adult ADHD patients analyzed with methods from graph theory
Source: PLoS One. 2020 Nov 9;15(11):e0241991. doi: 10.1371/journal.pone.0241991 (PMC7652335; doi:10.1371/journal.pone.0241991)
Supplement: S8 Table — (DOCX) [file pone.0241991.s008.docx]

**S8 Table**

**Effect of gender on actigraphic registrations in the morning and evening, 360 min (18 – 24) using analysis of covariance (ANCOVA).**

| **Healthy controls** |
| --- |
| **N = 30** |
| **Mean F = 0.920 P = 0.341** |
| **SD (% of mean) F = 0.000 P = 0.986** |
| **RMSSD (% of mean) F = 0.142 P = 0.708** |
| **RMSSD/SD F = 0.906 P = 0.345** |
| **Edges F = 0.449 P = 0.506** |
| **Components F = 0.086 P = 0.771** |
| **Bridges F = 1.494 P = 0.227** |
| **Missing edges F = 0.499 P = 0.483** |
| **Max number of edges F = 0.077 P = 0.783** |
| **Nodes with zero edges F = 0.045 P = 0.832** |
| **Ln cliques F = 0.001 P = 0.971** |
| **Sample entropy F = 1.607 P = 0.210** |
