# Supplementary material for: Diurnal variation of motor activity in adult ADHD patients analyzed with methods from graph theory
Source: PLoS One. 2020 Nov 9;15(11):e0241991. doi: 10.1371/journal.pone.0241991 (PMC7652335; doi:10.1371/journal.pone.0241991)
Supplement: S9 Table — Healthy controls, results for males and females separately. (DOCX) [file pone.0241991.s009.docx]

**S9 Table**

**Actigraphic registrations in the morning and evening, 360 min (08 – 14 and 18 - 24). Healthy controls, results for males and females separately.**

| **Males (n = 10) Females (n = 20)** |
| --- |
| **Morning Evening P Morning Evening P** |
|  |
| **Mean 332 ±111 305 ±101 0.576 411 ± 201 311 ± 158 0.087** |
| **SD 117 ± 29 125 ± 35 0.591 99 ± 31 143 ± 43 0.001** |
| **RMSSD 92 ± 17 94 ± 22 0.818 83 ± 17 109 ± 34 0.005** |
| **RMSSD/SD 0.81 ± 0.13 0.76 ± 0.08 0.411 0.87 ± 0.12 0.76 ± 0.09 0.002** |
| **Edges 6.88 ± 2.40 6.67 ± 2.26 0.791 8.48 ± 2.88 6.06 ± 2.71 0.019** |
| **Components 116 ± 38 128 ± 48 0.552 98 ± 33 155 ± 61 0.001** |
| **Bridges 38.8 ± 4.9 29.4 ± 7.2 0.003 38.5 ± 10.0 23.0 ± 12.8 <0.001** |
| **Missing edges 313 ± 14 318 ± 15 0.495 307 ± 15 317 ± 20 0.088** |
| **Max edges 20.9 ± 5.2 22.0 ± 7.2 0.701 22.3 ± 5.2 21.7 ± 8.1 0.781** |
| **Zero edges 126 ± 39 129 ± 39 0.878 112 ± 31 148± 55 0.015** |
| **Ln cliques 7. 52 ± 0.66 7.49 ± 0.70 0.936 7.77 ± 0.69 7.26 ± 0.94 0.054** |
| **Sample entropy 0.70 ± 0.33 0.63 ± 0.29 0.582 1.13 ± 0.45 0.46 ± 0.26 <0.001** |

Registrations in the morning and evening are compared with paired samples t-tests
