# Supplementary material for: Diurnal variation of motor activity in adult ADHD patients analyzed with methods from graph theory
Source: PLoS One. 2020 Nov 9;15(11):e0241991. doi: 10.1371/journal.pone.0241991 (PMC7652335; doi:10.1371/journal.pone.0241991)
Supplement: S10 Table — (DOCX) [file pone.0241991.s010.docx]

**S10 Table**

**Effect of gender on actigraphic registrations in the morning and evening, 360 min (18 – 24) using analysis of covariance (ANCOVA).**

| **ADHD** |
| --- |
| **N = 42 (morning) and 41 (evening)** |
| **Mean F = 1.244 P = 0.268** |
| **SD (% of mean) F = 4.617 P = 0.035** |
| **RMSSD (% of mean) F = 3.674 P = 0.059** |
| **RMSSD/SD F = 0.424 P = 0.517** |
| **Edges F = 6.155 P = 0.015** |
| **Components F = 4.594 P = 0.035** |
| **Bridges F = 2.145 P = 0.147** |
| **Missing edges F = 2.528 P = 0.116** |
| **Max number of edges F = 3.547 P = 0.063** |
| **Nodes with zero edges F = 2.809 P = 0.098** |
| **Ln cliques F = 3.478 P = 0.066** |
| **Sample entropy F = 4.247 P = 0.043** |
