# Supplementary material for: Diurnal variation of motor activity in adult ADHD patients analyzed with methods from graph theory
Source: PLoS One. 2020 Nov 9;15(11):e0241991. doi: 10.1371/journal.pone.0241991 (PMC7652335; doi:10.1371/journal.pone.0241991)
Supplement: S11 Table — ADHD patients. (DOCX) [file pone.0241991.s011.docx]

**S11 Table**

**Actigraphic registrations in the morning and evening, 360 min (08 – 14 and 18 - 24). ADHD patients.**

| **Males (n = 24)* Females (n = 18)** |
| --- |
| **Morning Evening P Morning Evening P** |
|  |
| **Mean 293 ± 160 330 ±197 0.477 283 ± 146 259 ± 127 0.602** |
| **SD 126 ± 49 121 ± 40 0.721 138 ± 49 150 ± 38 0.408** |
| **RMSSD 107 ± 41 100 ± 29 0.489 113 ± 33 124 ± 37 0.337** |
| **RMSSD/SD 0.87 ± 0.17 0.85 ± 0.12 0.568 0.85 ± 0.14 0.83 ± 0.15 0.746** |
| **Edges 6.73 ± 2.81 7.31 ± 2.83 0.486 6.12 ± 3.25 4.89 ± 1.98 0.141** |
| **Components 125 ± 67 125 ± 49 0.998 148 ± 68 159 ± 56 0.583** |
| **Bridges 37.9 ± 9.7 28.0 ± 10.5 0.002 33.6 ± 13.4 25.2 ± 11.4 0.052** |
| **Missing edges 320 ± 16 317 ± 13 0.484 322 ± 18 326 ± 14 0.508** |
| **Max edges 21.3 ± 6.4 21.9 ± 5.0 0.751 20.1 ± 8.1 17.9 ± 5.5 0.353** |
| **Zero edges 137 ± 63 130 ± 41 0.688 153 ± 60 154 ± 50 0.978** |
| **Ln cliques 7. 41 ± 0.84 7.55 ± 0.69 0.551 7.20 ± 0.97 6.87 ± 0.77 0.271** |
| **Sample entropy 0.81 ± 0.43 0.78 ± 0.48 0.805 0.70 ± 0.48 0.51 ± 0.25 0.161** |

*Morning, n = 24, evening, n =23

Registrations in the morning and evening are compared with paired samples t-tests
