# Supplementary material for: Diurnal variation of motor activity in adult ADHD patients analyzed with methods from graph theory
Source: PLoS One. 2020 Nov 9;15(11):e0241991. doi: 10.1371/journal.pone.0241991 (PMC7652335; doi:10.1371/journal.pone.0241991)
Supplement: Attachment — Submitted filename: fasmer_response to reviewers.docx [file pone.0241991.s013.docx]

**Response to Reviewers**

**Reviewer # 1 :**

1. Can authors declare the difference between this paper and Ref.17

**Response :**

*In Reference 17 (Fasmer OB, Mjeldheim K, Førland W, Hansen AL, Syrstad VEG, Oedegaard KJ, et al. Linear and non-linear analyses of Conner’s Continuous Performance Test-II discriminate adult patients with attention deficit hyperactivity disorder from patients with mood and anxiety disorders), we did use the same patient sample as in the present study, and furthermore divided tha sample into patients with and without ADHD. However, in Reference 17 we have looked at time series from the the CPT-test, and not from motor activity. In addition, cyclothymic temperament was a cental focus in that paper. In the present paper, besides using time series of motor activity, we study diurnal variation and used methods from graph theory to analyze the time series, in addtion to mathematical methods also utilized in reference 17.*

2.It is really hard for me to understand how a graph is built from a
single motor series

**Response :**

*Development of our similarity graph algorithm is based on work done by Lacasa et al (reference 23, Lacasa L, Luque B, Ballesteros F, Luque J, Nuño JC. From time series to complex networks: The visibility graph), where the theoretical background for transforming time series into graphs is described.*

*Motor activity time series is only one example of time series that can be trasformed into graphs, and in such a graph every node corresponds to a point in the time series, and two nodes are connected if the values of the motor activity are similar.*

*To explain in more detail : The similarity graph algorithm transforms time series into undirected similarity graphs, were two time points within a certain distance k connects with an edge if they correspond to each other within a given tolerance. The k preceding and the k succeeding time points of a random point in the time series defines to be nearest neighbors of such a time point. The threshold for similarity between two time points (u and v) defines as max (x_u_, x_v_) / min (x_u_, x_v_) < 1.2. Any time point with few or no connections (edges) to its nearest neighbors indicates an alteration in activity*

3.Discussion needs to be majorly revised. Many descriptions are actually
results (e.g., lines 452-456, 464-468). And many sentences are meaningless
(e.g., lines 448, 461-463). Most importantly, many measures can
characterize the diurnal difference, but their meanings for patients are
not clear explained. For example, in lines 425-426, the description on
RMSSD is actually a definition, not its meaning for ADHD. I suggest authors
pay more effort to dig deeper meaning of this paper.

**Response :**

*We have altered the parts of the discussion mentioned.*

*Concerning lines 425-426 : This must be seen in relation to the rest of this paragraph and the next. We agree with the reviewer that these parts need to be revised, and have made alterations from line 425 to line 440.*

*Concerning line 448: We have deleted the sentence that starts in line 448.*

*Concerning lines 452-456: We have made several alterations to this part.*

*Concerning lines 461-463: We have altered this sentence, and moved it some lines up in the same paragraph, hopefully making the meaning clearer.*

*Concerning lines 464-468:We have deleted the listing of results in the last sentence of this paragraph.*

*Concerning the deeper meaning of this paper: The changes we have made may hopefully give more meaning to the results we have presented. However, as we have stated in the conclusion section of the discussion, the present findings do not in any way fully explain the underlying dynamics of the diurnal regulation of motor activity, and activation in general. However, we think the present study may indicate the way foreward, namely using actigraph registrations in natural situations combined with mathematical methods to further unravel these mechanisms.*

4.I notice that many results are not provided in a table (e.g., lines
393-398) and just written in main text. I suggest that a summarize on
results in a table is more clear for readers.

**Response :**

*We agree that this would be clearer if presented in tables, and this has been done, by adding four more supplemental tables, and removing results from these paragraphs.*

*When writing these tables we have corrected some of the results (there are no significant effects of gender in the morning/evening comparisons for the healthy controls, and only four for the ADHD patients).*

*Concerning the effects of gender we have added a cautionary note with regard to the statistical results, noting that particularly for the healthy controls the groups are rather small, making the results more uncertain than for the groups containing both genders.*

5.In graph theory, many measures are given, but the degree, path, acyclic
and spanning subgraph are not used in later analysis. If true, please
delete them.

**Response :**

*We have altered the method section and removed the measures not used in the analyses. The definition of path is needed to define connected components and bridge. The definition of degree is needed to complete the definition of graph. We have however deleted the definition of tree and spanning graph.*

6.CT is cyclothymic temperament? Please identify the abbreviation while
using it first time.

**Response :**

*Yes, CT is cyclothymic temperament. We have identified this abbreviation the first time it was used (line 312).*

7.Line 323, 39 should be 39 patients or something.

**Response :**

*This has been amended to 39 patients.*

8.The grammatical tense is not consistent, e.g., lines338 uses 'was', line
342 uses 'is', pleases unite them through the paper.

**Response :**

*This has been altered, so the grammatical tense should now be consistent (past tense).*

9.Line 344, what does the (1.34) mean.

**Response :**

*This value is Cohens d, the sentence has been altered to make this clearer.*

10.After tables, *p=0.05 should be p<0.05.

**Response :**

*This has been altered.*

11.Line 385, p=0.05 is not significant, pleases revise the description

**Response :**

*This has been altered.*

12.Lines 397-398, I didn't found the results corresponding to the
description.

**Response :**

*The results are now presented in the supplemental tables 6 and 7.*

13.Line 404, I know that 8/12 indicates only 8 measures are significant,
but it is really a strange description, can we just use 8?

**Response :**

*We agree, and this has been altered.*

14.Line 483, authors should present the current results and then compare
them to previous.

**Response :**

*We have altered this part of the discussion and presented the results in more detail.*

15.Line 500, is it a replicated description 'differently put; reduced
amplitude….. Reduced….'

**Response :**

*Yes, it is just a different description of the same phenomenon, but we have altered the wording to : “or in other words”, to make this clearer..*

16.In conclusion, I suggest authors don't use newly, because it is hard to
identify what is really new.

**Response :**

*We have omitted this.*

17.Finally, can author explain to me why we have a higher variability and
lower complexity in the evening, does it relate to our brain in some
aspects?

**Response :**

*We have no clear explanation of this difference between morning and evening, but the inverse relation between variability and complexity is seen in different studies. See also response to reviewer 2.*

**Reviewer # 2 :**

Major 1: During the construction of similarity graph, the similar was
detected by max(ux,uv)/min(ux,uv)<1.2. The authors explained this
definition with analogy to the tolerance in sample entropy. However, unlike
sample entropy where the tolerance range is a fix value (0.2*std), this
definition will lead to a flexible range depending on the amplitude of the
denominator. In other word, smaller signal value in the bottom will have
narrower tolerance range and harder to "be similar" to others. The authors
should explain why they use this criteria and what will be the consequence
in the following computation of graph measures.

**Response :**

*We have tried to explain this a bit more detailed in the method section of the paper. It is correct that with our definition of similarity the tolerance for similarity will depend on the value for the points in the time series in the neighbourhood of the index point, but* *we think it is more reasonable to use such a criterion compared to using a fixed value for the whole series (as in sample entropy). One problem with sample entropy is that it is sensitive to outliers, which will increase SD and thus increase the probability that two points are considered similar, giving a false impression of high regularity, and a general problem with motor activity studies with actigraphs is that SD is high (and sometimes very high). We therefore think it makes sense to let the tolerance range for similarity depend on the actual values of the points to be compared, rather than to be dependent on all the values of the series (that is on the SD). The consequence will be that number of points considered similar will be lower, thus avoiding ceiling effects, and probably increase the ability of the test to detect differences between groups. This will be the case for all the graph measures we use.*

Minor 1: For readability, I suggest the authors to give a schematic
illustration for the construction of similarity graph on time-series. In
addition, authors can show example of actigraphs time-series. In addition,
different kinds of activity jumps detected by graph measures, degree,
bridge, and k-clique detect should be given to guide understanding.

**Response :**

*We have added an illustration of a similarity graph (Fig 2) and an example of an actigraph time series (Fig 1). There are two kind of “jumps” detected by the graph analyses, components and bridges, these are illustated in Fig 2. In addition the figure also illustrates the concept of clique.*

Minor 2: The discussion about diurnal fluctuations and weak circadian
rhythms, etc are too far away from the topic and the presented evidences,
given the fact that the authors cannot explain what is told by the bridge
changes.

**Response :**

*We have substantially shortened and rewritten this part of the discussion.*

Minor 3: Not clear why in healthy controls the evening registrations showed
higher variability and lower complexity compared to morning registrations.
authors can recheck.

**Response :**

*We do not have a ready explanation of why there is such a difference between morning and evening registrations. Obviously we need more information on the diurnal regulation of motor activity and activation in general to understand we this is so.*

*As we have noted in the response to reviewer 1, an inverse relation is seen between measures of variability and complexity is found also in other studies, for instance in a previous actigraph study from our group (Hauge ER, Berle JØ, Oedegaard KJ, Holsten F, Fasmer OB.* [*Nonlinear analysis of motor activity shows differences between schizophrenia and depression: a study using Fourier analysis and sample entropy.*](http://www.ncbi.nlm.nih.gov/pubmed/21297977) *PLoS One. 2011, 6 : e16291) and in a study on heart rate variability (Fasmer OB, Liao H, Huang Y, Berle JØ, Wu J, Oedegaard KJ, Wik G, Zhang Z.* [*A naturalistic study of the effect of acupuncture on heart-rate variability.*](http://www.ncbi.nlm.nih.gov/pubmed/22309903) *J Acupunct Meridian Stud. 2012, 5 : 15-20). The definition of these concepts (SD and sample entropy) are clearly different, but as this study also shows they are in some way related.*
